# Supplementary figures and images for: Folding–function relationship of the most common cystic fibrosis–causing CFTR conductance mutants
Source: Life Sci Alliance. 2019 Jan 18;2(1):e201800172. doi: 10.26508/lsa.201800172 (PMC6339265; doi:10.26508/lsa.201800172)

**Figure 3B**  
**Full gels**

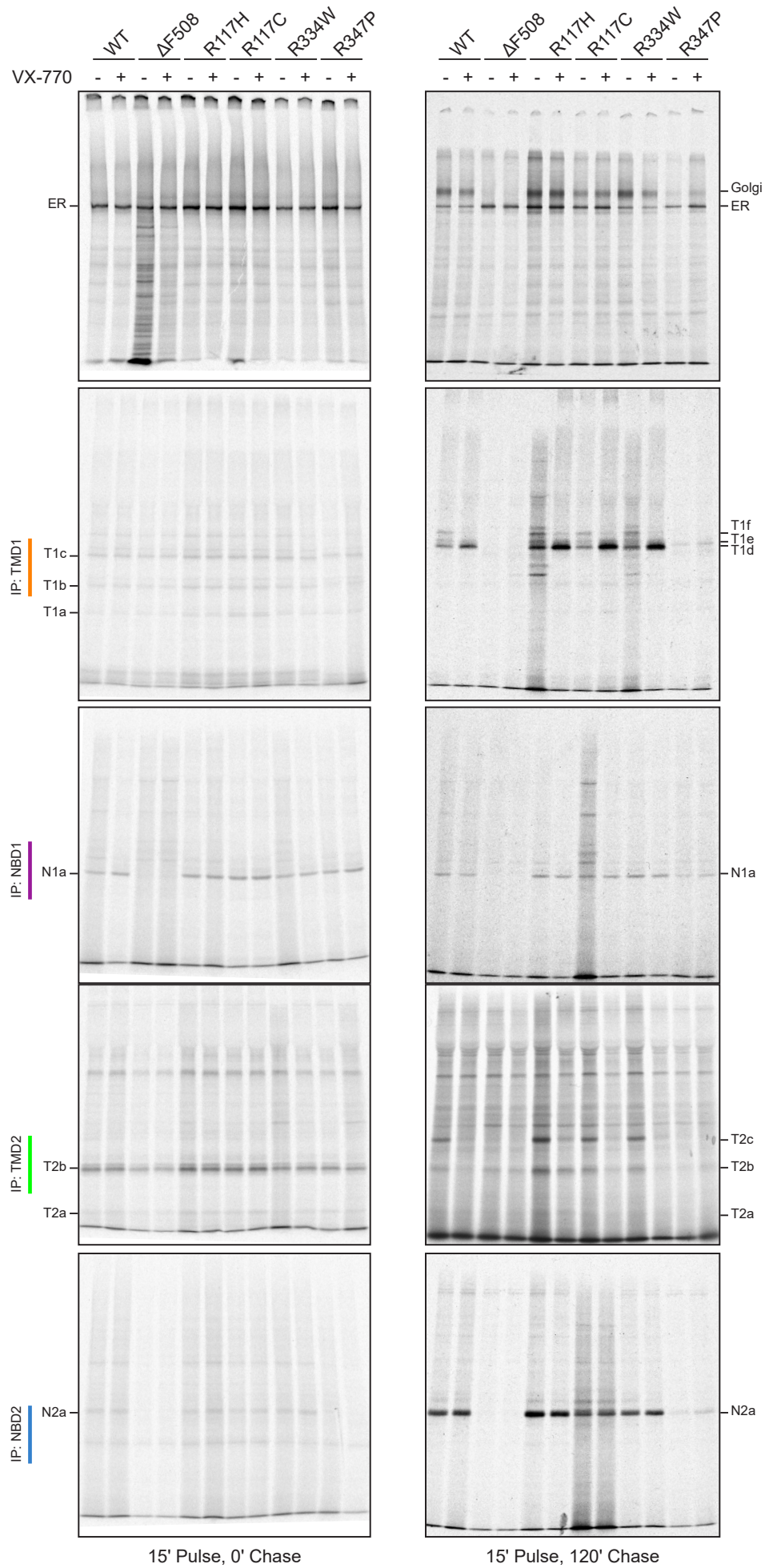

Supplement: Supplementary file 1 [file LSA-2018-00172_SdataF3.pdf]

**Figure 4a**  
**Full gels**

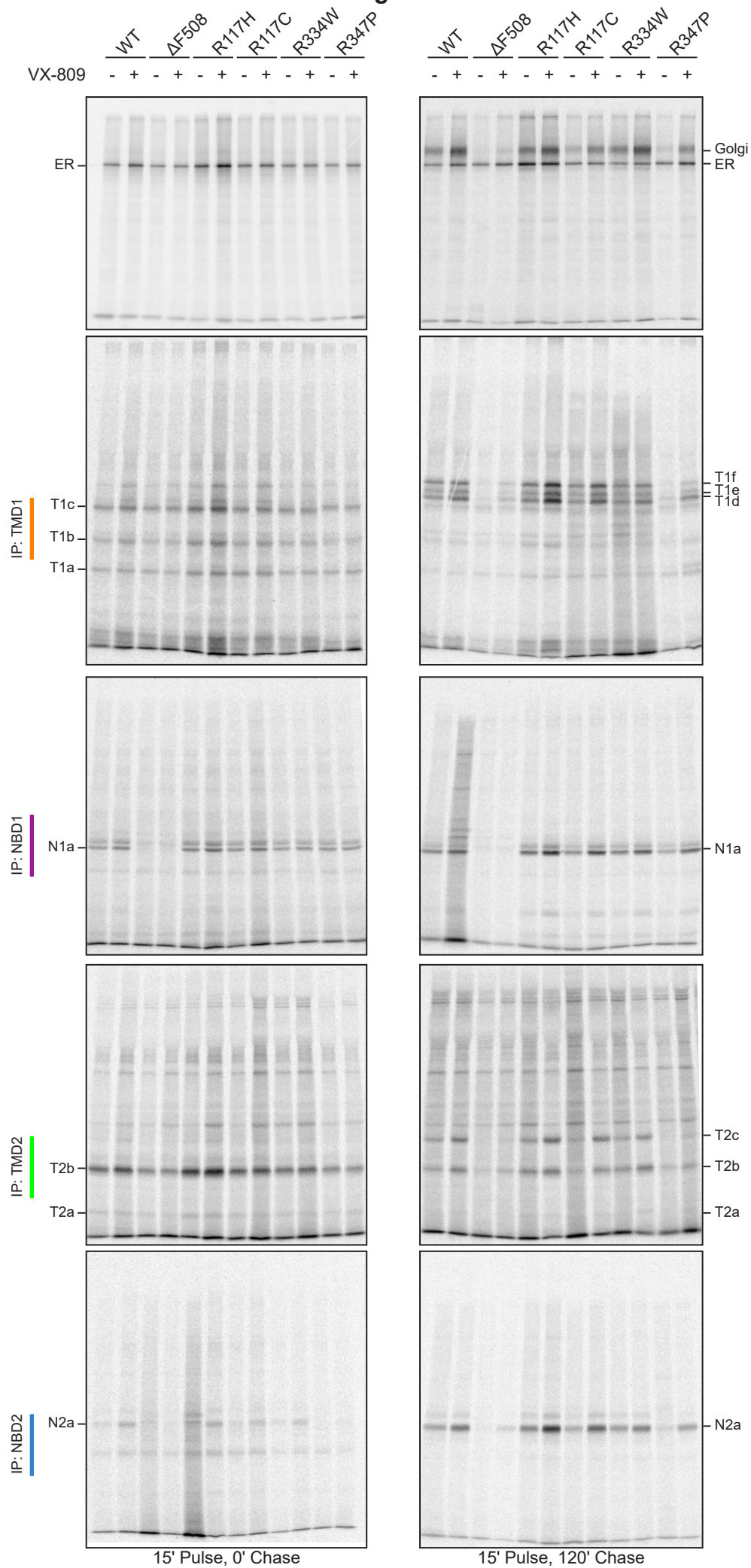

**Figure 4b**  
**Full gels**

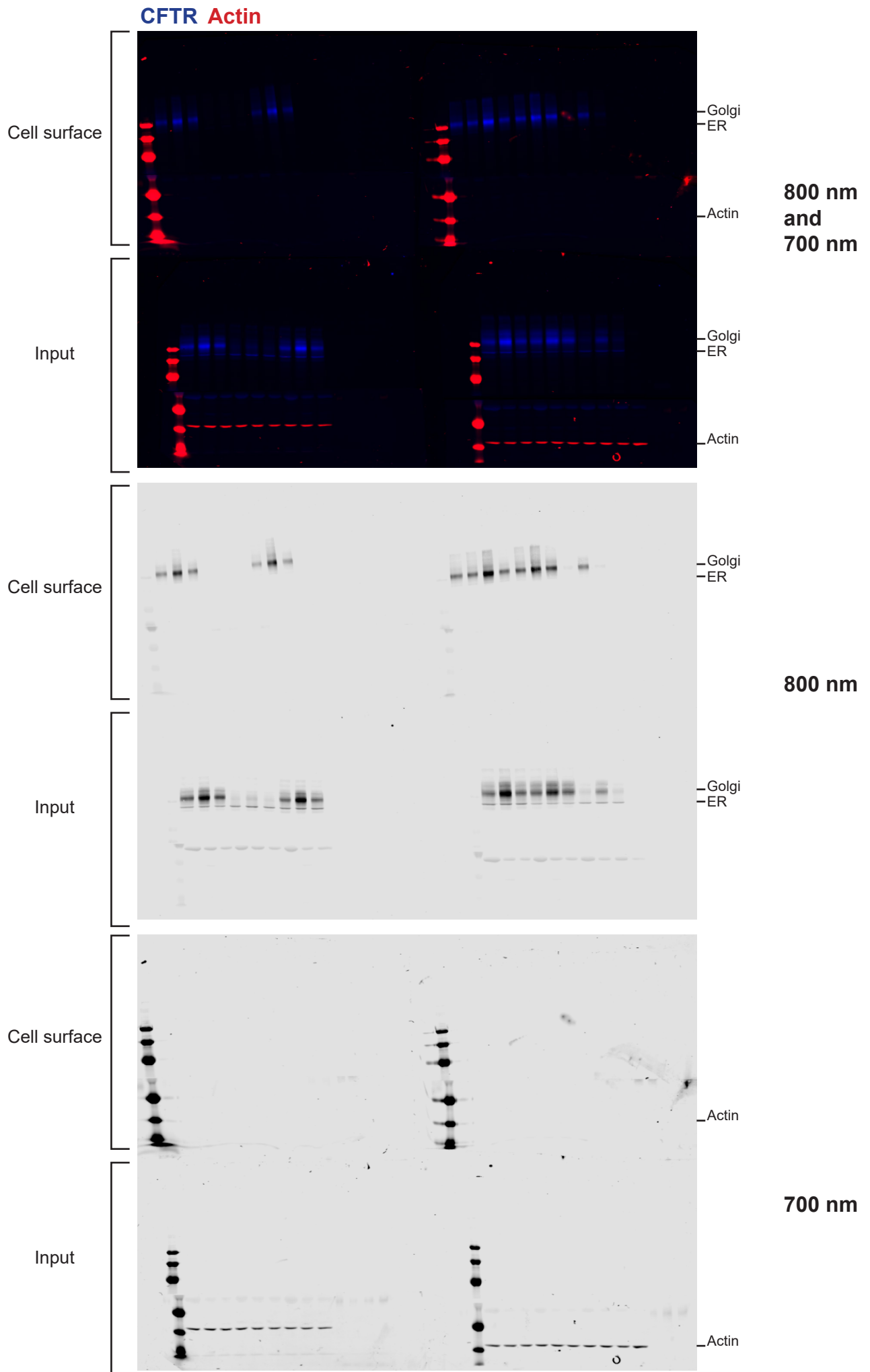

Figure 4C  
Full gels

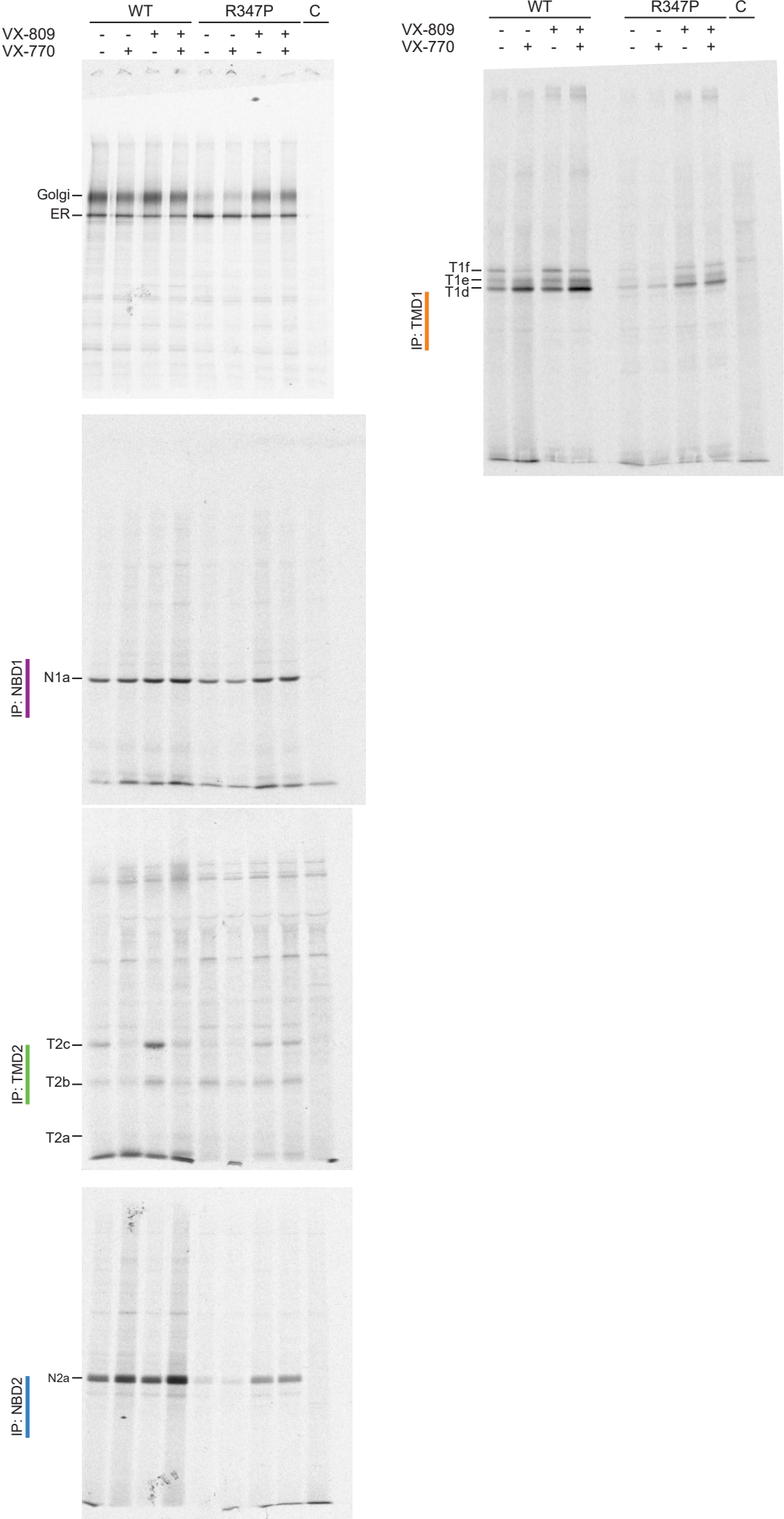

Supplement: Supplementary file 2 [file LSA-2018-00172_SdataF4.pdf]

Figure 5a  
Full gels

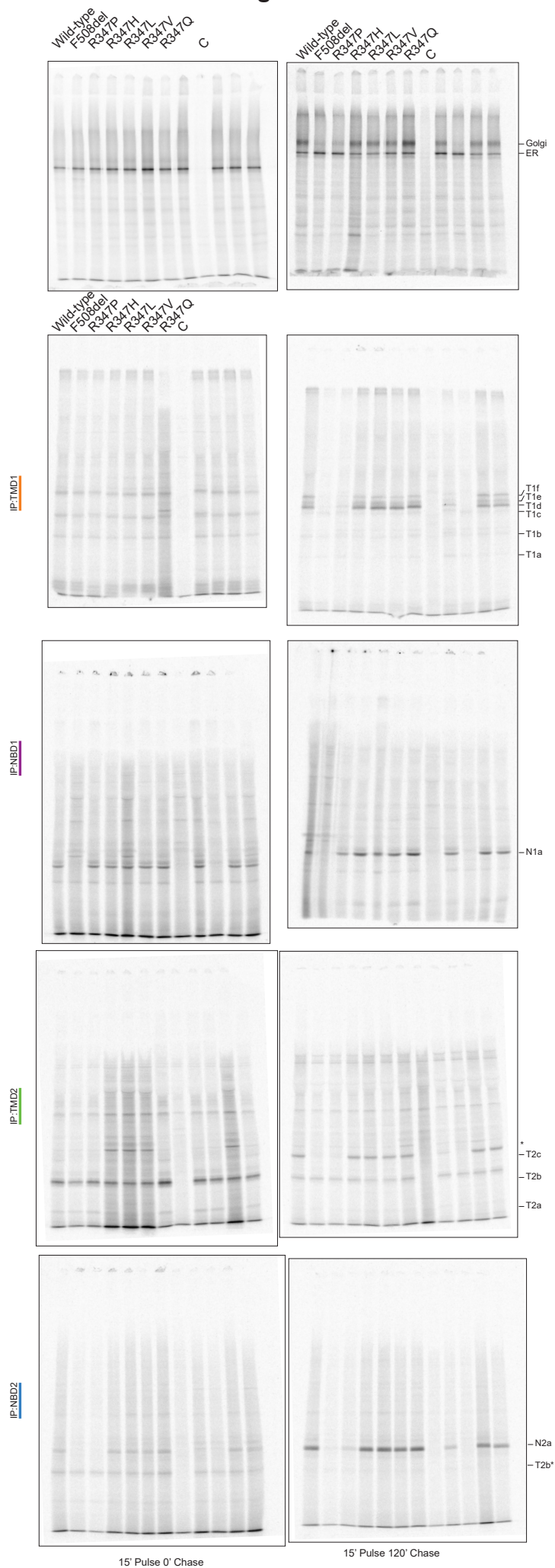

Supplement: Supplementary file 3 [file LSA-2018-00172_SdataF5.pdf]
